# The NSs protein of severe fever with thrombocytopenia syndrome virus differentially inhibits the type 1 interferon response among animal species

**DOI:** 10.1016/j.jbc.2023.104819

**Published:** 2023-05-13

**Authors:** Rokusuke Yoshikawa, Masahiro Kawakami, Jiro Yasuda

**Affiliations:** 1 Department of Emerging Infectious Diseases, Institute of Tropical Medicine (NEKKEN), Nagasaki University, Nagasaki, Japan; 2 Department of Emerging Infectious Diseases, National Research Center for the Control and Prevention of Infectious Diseases (CCPID), Nagasaki University, Nagasaki, Japan; 3 Graduate School of Biomedical Sciences and Program for Nurturing Global Leaders in Tropical and Emerging Communicable Diseases, Nagasaki University, Nagasaki, Japan

**Keywords:** animal virus, SFTSV, NSs, interferon, animal, signal transducers and activators of transcription 1, STAT2, innate immunity, negative-strand RNA virus

## Abstract

Severe fever with thrombocytopenia syndrome virus (SFTSV), which has been reported in China, Korea, Japan, Vietnam, and Taiwan, is a causative agent of severe fever thrombocytopenia syndrome. This virus has a high mortality and induces thrombocytopenia and leukocytopenia in humans, cats, and aged ferrets, whereas immunocompetent adult mice infected with SFTSV never show symptoms. Anti-SFTSV antibodies have been detected in several animals—including goats, sheep, cattle, and pigs. However, there are no reports of severe fever thrombocytopenia syndrome in these animals. Previous studies have reported that the nonstructural protein NSs of SFTSV inhibits the type I interferon (IFN-I) response through the sequestration of human signal transducer and activator of transcription (STAT) proteins. In this study, comparative analysis of the function of NSs as IFN antagonists in human, cat, dog, ferret, mouse, and pig cells revealed a correlation between pathogenicity of SFTSV and the function of NSs in each animal. Furthermore, we found that the inhibition of IFN-I signaling and phosphorylation of STAT1 and STAT2 by NSs depended on the binding ability of NSs to STAT1 and STAT2. Our results imply that the function of NSs in antagonizing STAT2 determines the species-specific pathogenicity of SFTSV.

Severe fever with thrombocytopenia syndrome (SFTS) is an emerging infectious disease caused by the SFTS virus (SFTSV), a novel Bandavirus belonging to the order Bunyavirales, in the family Phenuiviridae. SFTS has been reported in Central China, South Korea, Japan, Vietnam, and Taiwan (1, 2, 3, 4, 5, 6). In humans, SFTS is characterized by fever, diarrhea, vomiting, thrombocytopenia, leukocytopenia, and elevated liver enzyme levels (7, 8, 9). Recently, cheetahs in a zoo and domestic cats and dogs were also diagnosed with SFTS in Japan and South Korea (10, 11, 12, 13, 14). By 2019, case reports of SFTS in 120 cats and 7 dogs had been published. The fatality rates of cats and dogs in Japan were approximately 60 to 70% and 29%, respectively (https://www.niid.go.jp/niid/ja/allarticles/surveillance/2467-iasr/related-articles/related-articles-473/8988-473r06.html). In addition, SFTSV is highly lethal to aged ferrets in experimental infections (15), whereas it is weakly pathogenic to rodents and hamsters (16, 17). Although previous epidemiological studies reported the detection of anti-SFTSV antibodies (immunoglobulin M and immunoglobulin G) in goats, sheep, cattle, and pigs, the pathogenicity of SFTSV in these animals is considered low (18). Thus, the pathogenicity of SFTSV varies among animals, although the underlying mechanism remains unclear.

The genome of SFTSV consists of three negative-stranded large (L), medium (M), and small (S) RNA segments. The L and M segments encode the viral RNA–dependent RNA polymerase and envelope proteins, respectively. The S segment encodes the nucleocapsid protein (N) and the nonstructural protein (NSs).

The type I interferon (IFN-I) response (an innate immune response) is induced by viral infection and prevents viral replication (19). Antiviral innate immunity is initiated by the binding of viral RNA to cellular pattern recognition receptors such as retinoic acid–inducible gene I–like receptors. Binding of viral RNA to retinoic acid–inducible gene I–like receptors results in the activation of interferon regulatory factor-3 (IRF-3) through TANK-binding kinase 1 (TBK1). Activated IRF-3 acts as a transcription factor for IFN-I induction. The binding of secreted IFN to IFN receptors results in phosphorylation of signal transducer and activator of transcription 1 (STAT1) and STAT2. The heterodimers or homodimers of phosphorylated STATs, together with IRF-9, form heterotrimeric IFN-stimulated gene factor 3 (ISGF3). The translocation of ISGF3 into the nucleus induces the activation of antiviral IFN-stimulated genes (ISGs) by binding to an IFN-stimulated response element (20).

Previous studies have reported that IFN-I induction and signaling are suppressed by NSs of SFTSV by binding to human TBK1, STAT1, and STAT2 proteins (21, 22, 23, 24). However, we have revealed that NSs cannot suppress IFN-I signaling in murine cells because of their inability to bind to murine STAT1 and STAT2 (24). Nevertheless, the activity of NSs in other animals that can be infected with SFTSV, such as cats, dogs, ferrets, and pigs, remains unknown.

To understand the relationship between SFTSV pathogenesis and the activity of NSs as IFN antagonists in various animal species, we compared the abilities of NSs as IFN antagonists in cells derived from several animal species.

## Results

### Comparison of the growth kinetics between SFTSV WT and SFTSV ΔNSs in cells derived from various animal species

**Figure 1:**
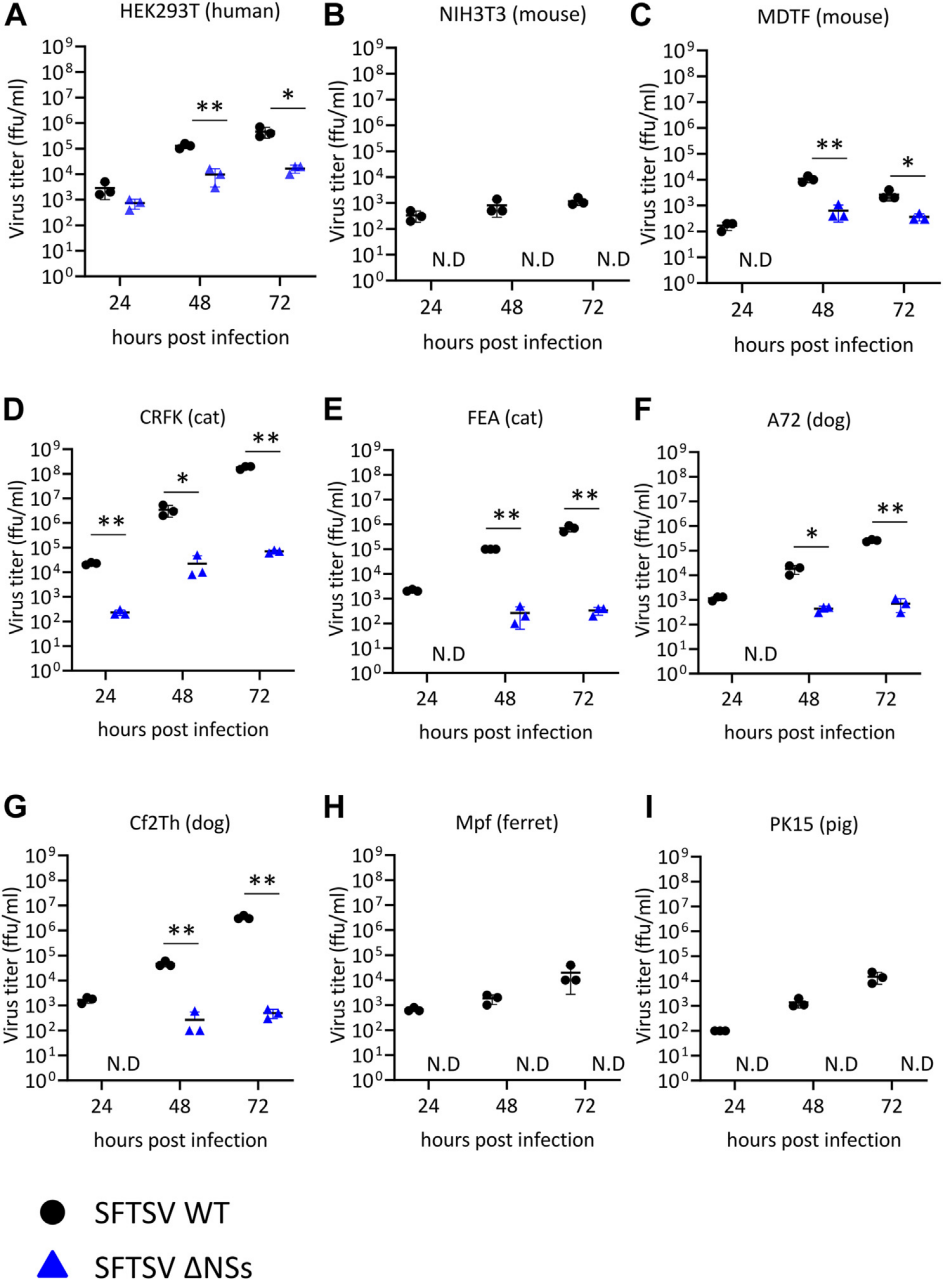
**Growth of SFTSV WT and SFTSV ΔNSs in cells derived from different animal species.***A*, HEK293T (human), (*B*) NIH3T3 (mouse), (*C*) MDTF (mouse), (*D*) CRFK (cat), (*E*) FEA (cat), (*F*) A72 (dog), (*G*) Cf2Th (dog), (*H*) Mpf (ferret), and (*I*) PK15 (pig) cells infected with either SFTSV WT or SFTSV ΔNSs at an MOI of 0.1. Each virus released in the culture supernatants was monitored by focus-forming assay. The *black dots* (●) and *blue triangles* () represent SFTSV WT and SFTSV ΔNSs, respectively. Values are the averages with SDs of data from three independent experiments (n = 3). ∗*p* < 0.05 and ∗∗*p* < 0.01, SFTSV WT infected *versus* SFTSV ΔNSs infected. Each exact *p* value, average, and SD is shown in Table S1. CRFK, Crandell–Rees feline kidney; HEK293T, human embryonic kidney 293T cell line; MOI, multiplicity of infection; ND, not detected; NSs, nonstructural protein; SFTSV, severe fever with thrombocytopenia syndrome virus.

The effect of SFTSV NSs on SFTSV replication in cell lines derived from several animal species was examined by generating WT and NSs-deficient SFTSV (SFTSV WT and SFTSV ΔNSs, respectively). These were generated using a reverse genetics system based on the SFTSV YG1 strain isolated from the first SFTS patient identified in Japan. Human embryonic kidney (HEK) 293T (human), NIH3T3 (mouse), *Mus dunni* tail fibroblast (MDTF) (mouse), Crandell–Rees feline kidney (CRFK) (cat), FEA (cat), A72 (dog), Cf2Th (dog), Mpf (ferret), and PK15 (pig) cells were infected with either SFTSV WT or SFTSV ΔNSs at a multiplicity of infection (MOI) of 0.1. The titers of SFTSV WT produced in HEK293T (human), NIH3T3 (mouse), MDTF (mouse), CRFK (cat), FEA (cat), A72 (dog), Cf2Th (dog), Mpf (ferret), and PK15 (pig) cells were 2.3 × 10^5^, 1.2 × 10^3^, 2.7 × 10^3^, 1.8 × 10^8^, 7.0 × 10^5^, 3.7 × 10^4^, 3.3 × 10^6^, 2.0 × 10^4^, and 1.5 × 10^4^ focus-forming units (FFU)/ml, respectively, at 72 h postinfection (hpi) (Fig. 1, *A*–*I*). The titers of SFTSV ΔNSs produced in HEK293T (human), MDTF (mouse), CRFK (cat), FEA (cat), A72 (dog), and Cf2Th (dog) cells were 1.7 × 10^4^, 2.0 × 10^2^, 7.0 × 10^4^, 3.3 × 10^2^, 3.3 × 10^3^, and 5.0 × 10^2^ FFU/ml, respectively, at 72 hpi (Fig. 1, *A*–*I*). However, we could not detect the production of SFTSV ΔNSs in NIH3T3 (mouse), Mpf (ferret), and PK15 (pig) cells. The replication efficacy of SFTSV ΔNSs varied among these cell lines. Consequently, the growth efficacy of SFTSV ΔNSs in each tested cell line was significantly lower than that of SFTSV WT (Fig. 1, *A*–*I*). Altogether, the results suggested that SFTSV NSs is an important factor required for the efficient replication of SFTSV, regardless of the cell line.

### Anti-IFN-I–inducing activities of NSs in cells derived from different animal species

**Figure 2:**
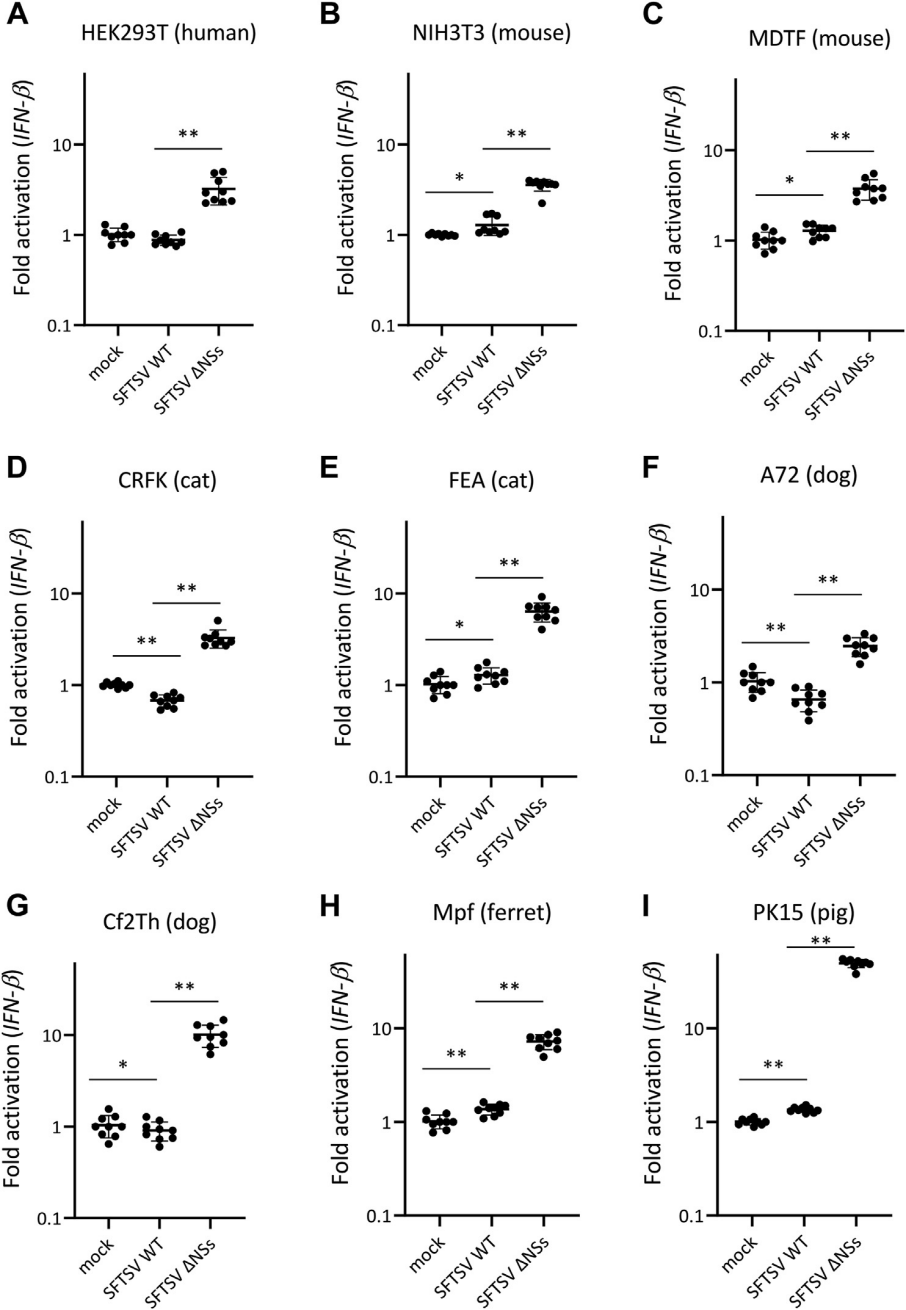
**Ability of NSs as an antagonist to IFN-I induction during infection in cells derived from different animal species.** (*A*) HEK293T (human), (*B*) NIH3T3 (mouse), (*C*) MDTF (mouse), (*D*) CRFK (cat), (*E*) FEA (cat), (*F*) A72 (dog), (*G*) Cf2Th (dog), (*H*) Mpf (ferret), and (*I*) PK15 (pig) cells were mock infected or infected with SFTSV WT or SFTSV ΔNSs at an MOI of 10. After 18 h, total RNA was extracted from these cells, and an mRNA expression level of IFN-β was quantified by real-time RT–PCR. The mRNA expression levels of IFN-β in mock-infected cells were set as 1. These assays were independently performed in triplicate. Values are the averages with SDs of data from nine results obtained from three experiments (n = 9). ∗*p* < 0.05, ∗∗*p* < 0.01, SFTSV WT infected *versus* SFTSV ΔNSs infected. Each exact *p* value, average, and SD is shown in Table S2. CRFK, Crandell–Rees feline kidney; HEK293T, human embryonic kidney 293T cell line; IFN-I, type I interferon; MDTF, *Mus dunni tail* fibroblast; MOI, multiplicity of infection; NSs, nonstructural protein; SFTSV, severe fever with thrombocytopenia syndrome virus.

To investigate whether NSs antagonizes IFN-I induction during SFTSV infection in cell lines derived from several animal species, HEK293T (human), NIH3T3 (mouse), MDTF (mouse), CRFK (cat), FEA (cat), A72 (dog), Cf2Th (dog), Mpf (ferret), and PK15 (pig) cells were infected with either SFTSV WT or SFTSV ΔNSs at an MOI of 10. At 18 hpi, mRNA expression of IFN-β, which is one of IFN-I, was quantified by real-time RT–PCR. In all tested cell lines, IFN-β induction by SFTSV ΔNSs was significantly stronger than that by SFTSV WT (Fig. 2). These results suggest that NSs inhibited the induction of IFN-I by SFTSV infection in these animals.

### Binding activity of NSs to each animal TBK1

**Figure 3:**
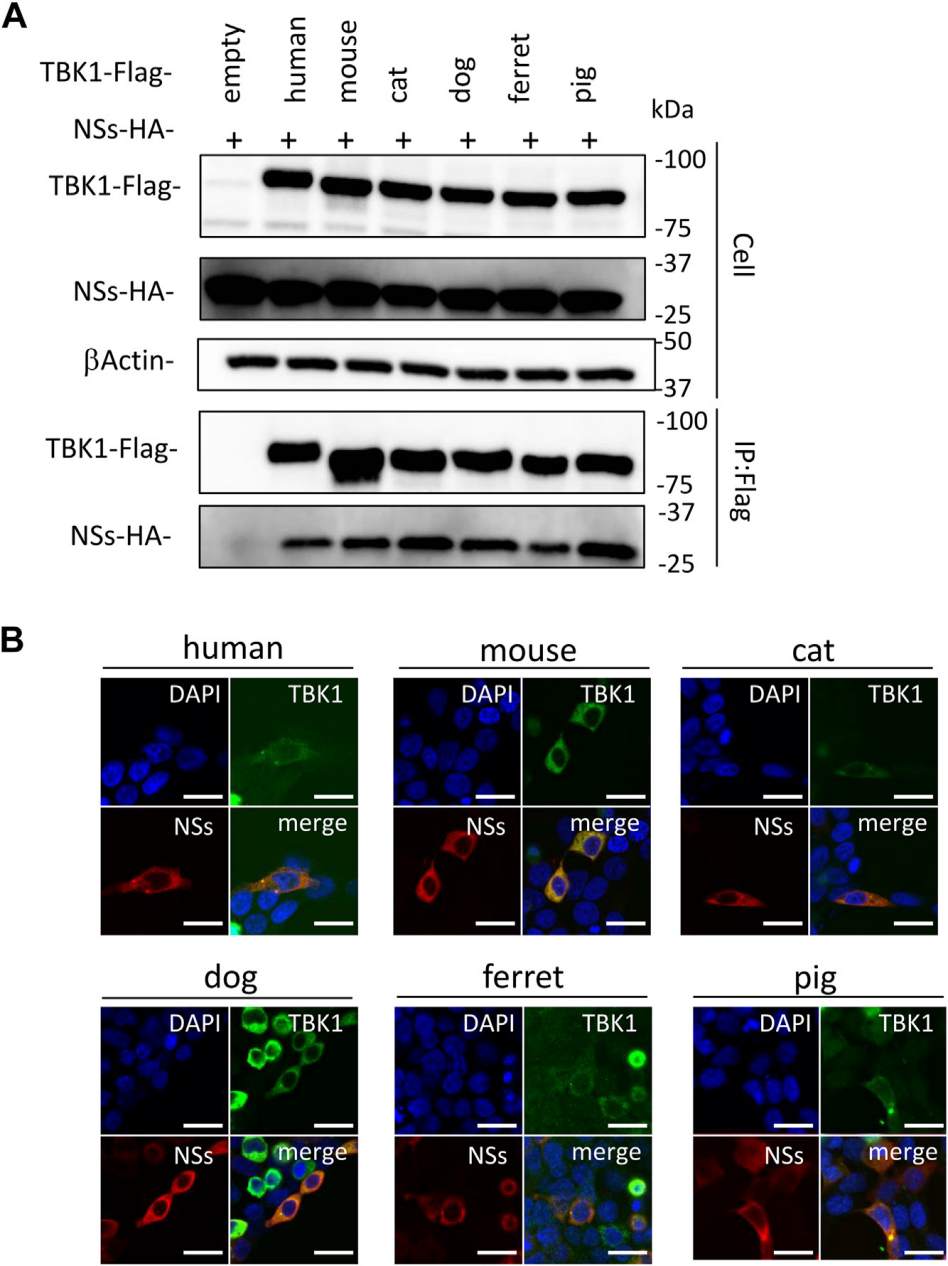
**Binding of NSs with TBK1.***A*, HEK293T (human) cells were transfected with the expression plasmid for HA-tagged NSs and each of the FLAG-tagged TBK1, and then co-IP assays were performed. *B*, colocalization of NSs with TBK1. HEK293T (human) cells were transfected with the expression plasmid for HA-tagged NSs and each of the FLAG-tagged TBK1. IFA was also performed with NSs, TBK1, and the nuclei, shown in *red*, *green*, and *blue*, respectively. Scale bar represents 20 μm. Representative results of Western blotting assays (*A*) and IFA (*B*) are shown. co-IP, coimmunoprecipitation; HA, hemagglutinin; HEK293T, human embryonic kidney 293T cell line; IFA, immunofluorescence assay; NSs, nonstructural protein; TBK1, TANK-binding kinase 1.

SFTSV NSs inhibits IFN-I inducing by interacting with TBK1 (23). Therefore, we investigated the binding activity of NSs to TBK1. To investigate the interaction of NSs with each animal TBK1, we performed coimmunoprecipitation (co-IP) assays using lysates from HEK293T (human) cells transfected with the expression plasmids for hemagglutinin (HA)-tagged NSs and each FLAG-tagged animal TBK1. As shown in Figure 3*A*, co-IP of all TBK1s with NSs was observed in IP elutes.

The interaction of NSs with TBK1 was also investigated by subcellular colocalization of the proteins using an indirect immunofluorescence assay (IFA). The NSs expression plasmid was cotransfected with the expression plasmids for human, murine, feline, canine, ferret, or porcine TBK1 into HEK293T cells. As shown in Figure 3*B*, colocalization of NSs with all TBK1s was observed. These findings indicate that NSs interacts with human, murine, feline, canine, ferret, and porcine TBK1.

### Anti-IFN-I signaling activities of NSs in cells derived from different animal species

**Figure 4:**
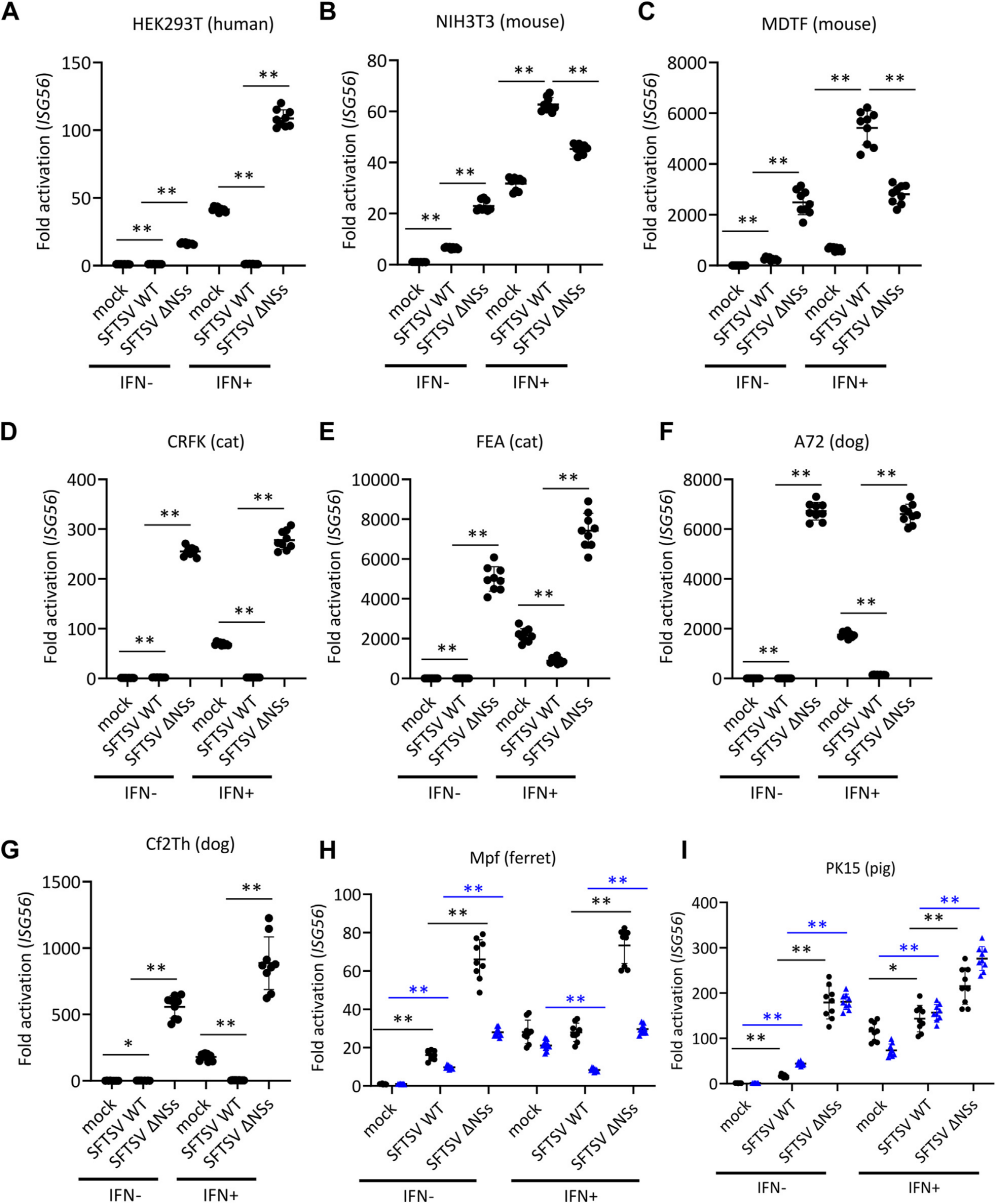
**Function of NSs as an antagonist to IFN-I signaling in cells derived from different animal species.***A*, HEK293T (human), (*B*) NIH3T3 (mouse), (*C*) MDTF (mouse), (*D*) CRFK (cat), (*E*) FEA (cat), (*F*) A72 (dog), (*G*) Cf2Th (dog), (*H*) Mpf (ferret), and (*I*) PK15 (pig) cells were mock infected or infected with SFTSV WT or SFTSV ΔNSs at an MOI of 10. After 24 or 48 h, the cells were treated with IFN-αA/D (500 U/ml) or left untreated for 18 h. Expression levels of *ISG56* mRNAs in each cell line were measured by real-time RT–PCR. The mRNA expression levels of *ISG56* in IFN-αA/D-untreated cells were set as 1. These assays were independently performed in triplicate. Values are the averages with SDs of data from nine results obtained from three experiments (n = 9). The *black* and *blue dots* show the result at 42 and 66 h after infection, respectively. ∗*p* < 0.05, ∗∗*p* < 0.01, *versus* SFTSV WT infected. Each exact *p* value, average, and SD is shown in Table S3. CRFK, Crandell–Rees feline kidney; HEK293T, human embryonic kidney 293T cell line; IFN-I, type I interferon; MDTF, *Mus dunni* tail fibroblast; MOI, multiplicity of infection; NSs, nonstructural protein; SFTSV, severe fever with thrombocytopenia syndrome virus.

To analyze the effect of NSs on IFN-I-induced ISG expression in cell lines derived from several animal species, HEK293T (human), NIH3T3 (mouse), MDTF (mouse), CRFK (cat), FEA (cat), A72 (dog), Cf2Th (dog), Mpf (ferret), and PK15 (pig) cells were infected with SFTSV WT or SFTSV ΔNSs at an MOI of 10. At 24 hpi, cells were treated with or without IFN-αA/D (500 U/ml) for 18 h, and then mRNA induction of ISG56, which is one of ISGs, was quantified using real-time RT–PCR. In all untreated cells, the expression of ISG56 mRNA in cells infected with SFTSV WT was weaker than that in cells infected with SFTSV ΔNSs, since NSs interfered with the induction of IFN-I during infection in these cell lines (Fig. 4, *A*–*H*). We analyzed the induction of ISG56 mRNA expression by IFN-I treatment. SFTSV WT, but not SFTSV ΔNSs, suppressed the induction of the *ISG56* gene by IFN-I in HEK293T (human), CRFK (cat), FEA (cat), A72 (dog), and Cf2Th (dog) cells (Fig. 3, *A*, *C*, *D*, *E*, and *F*), whereas both viruses showed little or no inhibition of ISG56 induction in NIH3T3 (mouse), MDTF (mouse), Mpf (ferret), and PK15 (pig) cells (Fig. 4, *B*, *G*, and *H*). Although SFTSV infection is lethal to aged ferrets, SFTSV WT could not inhibit the induction of ISG expression by IFN-I in Mpf (ferret) cells at 42 hpi. In addition, the replication efficiency of SFTSV WT in Mpf (ferret) and PK15 (pig) cells was lower than that in HEK293T (human), CRFK (cat), FEA (cat), A72 (dog), and Cf2Th (dog) cells (Fig. 1, *A*, *D*, *E*, *F*, *G*, and *H*). Therefore, Mpf (ferret) and PK15 (pig) cells were infected with SFTSV WT and SFTSV ΔNSs for 48 h and then treated with or without IFN-αA/D (500 U/ml) for 18 h (total 66 hpi). Induction of ISG56 by IFN-I in Mpf (ferret) cells was suppressed by SFTSV WT but not by SFTSV ΔNSs (Fig. 4*H*). However, in PK15 (pig) cells, SFTSV WT did not suppress IFN-I-induced ISG56 induction at 66 hpi (Fig. 4*I*). These results indicate that SFTSV NSs can suppress IFN-I signaling in feline, canine, ferret, and human cells, whereas SFTSV NSs cannot interfere with this signaling in porcine or murine cells. In addition, our studies suggest that NSs can suppress IFN-I induction during infection, regardless of the animal species, whereas the ability to suppress IFN-I signaling is species specific. Our findings suggest that the antagonistic effect of SFTSV NSs on IFN-I signaling is one of the factors that determine variation in SFTSV pathogenicity among animal species. Therefore, we further analyzed the mechanism underlying the anti-IFN-I signaling activity of NSs at the molecular level.

### Comparison of the phosphorylation of STAT1 and STAT2 induced by IFN-I in cells derived from different animal species

**Figure 5:**
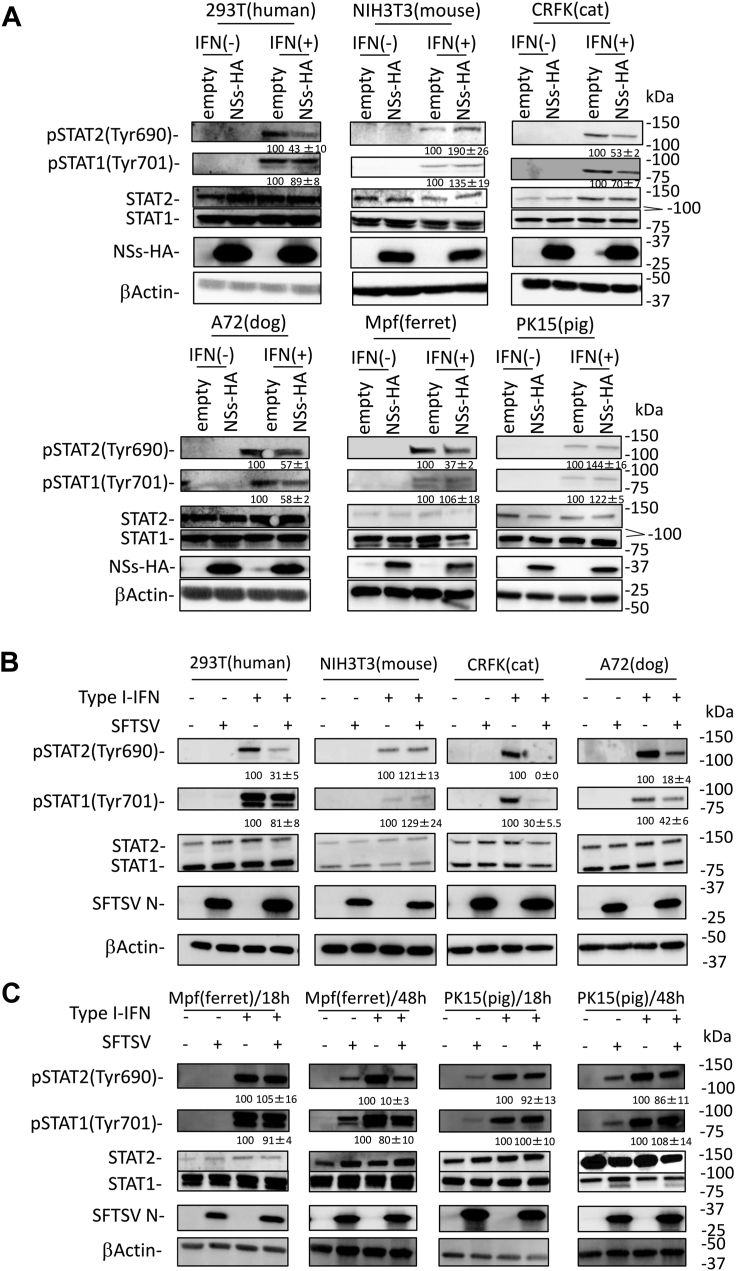
**Suppression of STAT1 and STAT2 phosphorylation by NSs.***A*, HEK293T (human), NIH3T3 (mouse), CRFK (cat), A72 (dog), Mpf (ferret), or PK15 (pig) cells transfected with expression plasmid for HA-tagged NSs were treated with IFN-αA/D (2000 U/ml) or left untreated for 30 min and were then lysed for detection of each protein expression by immunoblotting. *B* and *C*, HEK293T (human), NIH3T3 (mouse), CRFK (cat), A72 (dog), Mpf (ferret), or PK15 (pig) cells were infected with SFTSV at an MOI of 10. *C*, after 18 h or 48 h, these cells were treated with IFN-αA/D (2000 U/ml) or left untreated for 30 min and were then lysed for detection of each protein expression by immunoblotting. The relative expression rates of pSTAT1 and pSTAT2 in mock-transfected cells and uninfected cells treated with IFN-I are set at 100%. The assays were independently performed in triplicate. The data represent averages with SDs. CRFK, Crandell–Rees feline kidney; HA, hemagglutinin; HEK293T, human embryonic kidney 293T cell line; IFN, interferon; MOI, multiplicity of infection; NSs, nonstructural protein; SFTSV, severe fever with thrombocytopenia syndrome virus; STAT, signal transducer and activator of transcription.

Tyrosine phosphorylation of STAT1 and STAT2 is important for their function as transcription factors in the IFN-I signaling pathway (25). To examine the phosphorylation of STAT1 and STAT2 induced by IFN-I in cell lines derived from different animal species, HEK293T (human), NIH3T3 (mouse), CRFK (cat), A72 (dog), Mpf (ferret), and PK15 (pig) cells were transfected with or without the NSs-HA expression plasmid and then treated with IFN-αA/D (2000 U/ml) for 30 min. In all cell lines, the protein expression levels of STAT1 and STAT2 were stable, regardless of NSs expression (Fig. 5*A*). In the absence of NSs, IFN-I induced phosphorylation of both STAT1 and STAT2 in all cell lines. Consistent with our previous observation (24), NSs downregulated the phosphorylation of human STAT2 in HEK293T (human) cells by 33%, whereas no such effect was observed on murine STAT2 in NIH3T3 (mouse) cells (Fig. 5*A*). Under these conditions, NSs reduced the phosphorylation of feline, canine, and ferret STAT2 in CRFK (cat), A72 (dog), and Mpf (ferret) cells to 53%, 57%, and 37% of the mock level, respectively (Fig. 5*A*). In contrast, porcine STAT2 in PK15 (pig) cells was phosphorylated independently of the NSs expression (Fig. 5*A*). As reported previously (21, 23), NSs did not interfere with the phosphorylation of human STAT1 in HEK293T (human) cells (Fig. 5*A*). As shown in Figure 5*A*, the phosphorylation of murine, ferret, and porcine STAT1 in NIH3T3 (mouse), Mpf (ferret), and PK15 (pig) cells was not suppressed by NSs. The phosphorylation of feline and canine STAT1 in CRFK (cat) and A72 (dog) cells transfected with NSs reduced to 63% and 58%, respectively, compared with that in the mock-transfected CRFK (cat) and A72 (dog) cells (Fig. 5*A*). Phosphorylation of STAT1 and STAT2 was examined in SFTSV-infected cells. At 18 hpi, the phosphorylation level of human, feline, and canine STAT2 was 31%, 0%, and 18% in HEK293T (human), CRFK (cat), and A72 (dog) cells infected with SFTSV, respectively, compared with that in the mock-infected HEK293T (human), CRFK (cat), and A72 (dog) cells (Fig. 5*B*). In contrast, murine, ferret, and porcine STAT2 were phosphorylated independently of SFTSV infection in NIH3T3 (mouse), Mpf (ferret), and PK15 (pig) cells (Fig. 5*B*). However, NSs inhibited the phosphorylation of ferret STAT2 in Mpf (ferret) cells transfected with the NSs expression plasmid (Fig. 5*A*). Therefore, we examined the influence of SFTSV infection on the phosphorylation of ferret STAT2 in Mpf (ferret) cells at 48 hpi. IFN-I-induced phosphorylation of ferret STAT2 was reduced to 10% of the original level in the SFTSV-infected Mpf (ferret) cells (Fig. 5*B*). Analysis of the effect on STAT2 phosphorylation by SFTSV infection in PK15 (pig) cells at 48 hpi showed that porcine STAT2 was phosphorylated regardless of SFTSV-infection time (Fig. 5*B*). In addition, the effect of SFTSV infection on IFN-I-induced phosphorylation of STAT1 was similar to that of NSs transfection in HEK293T (human), NIH3T3 (mouse), A72 (dog), CRFK (cat), Mpf (ferret), and PK15 (pig) cells (Fig. 5, *A* and *B*).

**Figure 6:**
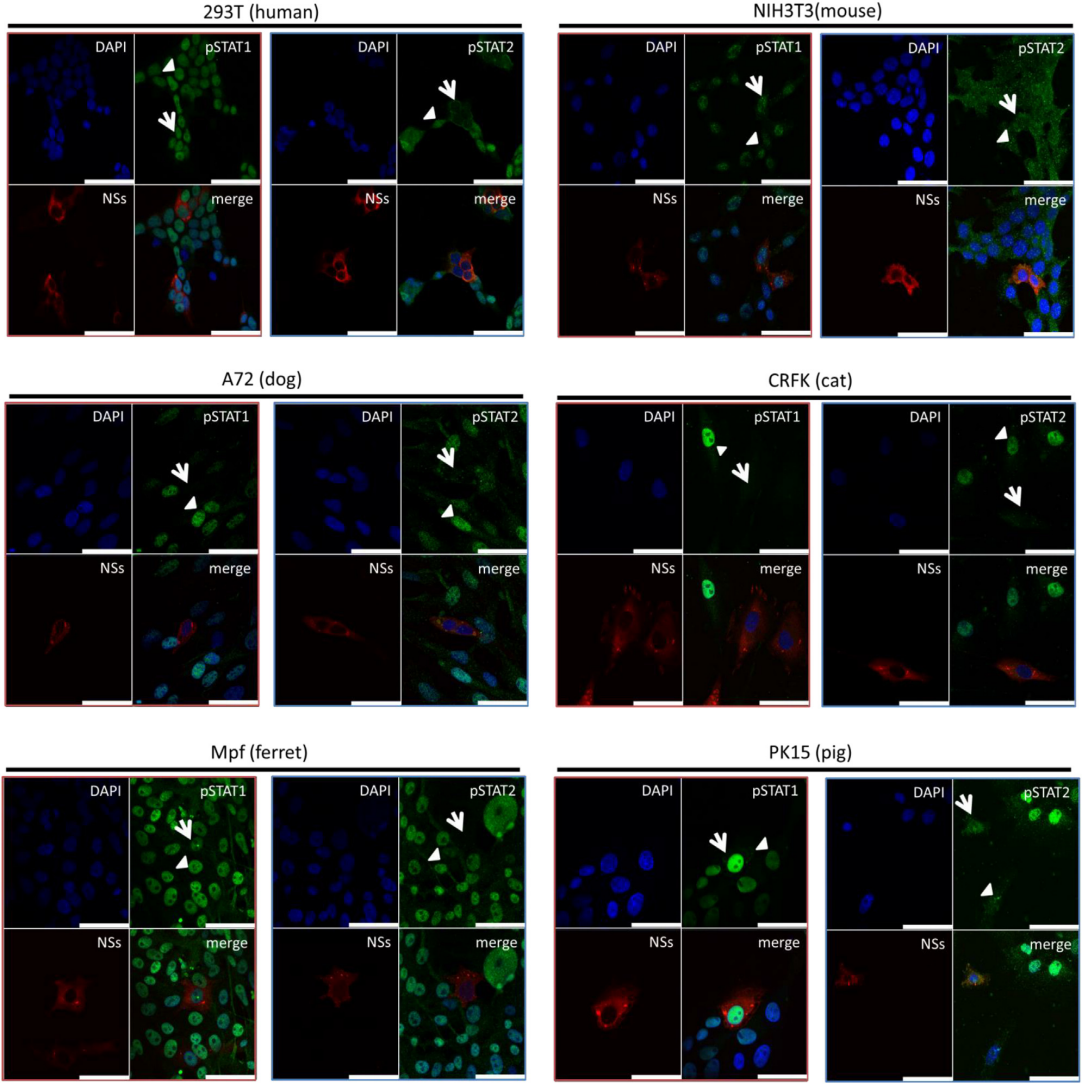
**Influence of NSs to STAT1 and STAT2 activation in cells derived from different animal species.** HEK293T (human), NIH3T3 (mouse), CRFK (cat), A72 (dog), Mpf (ferret), or PK15 (pig) cells were transfected with the expression plasmid for HA-tagged NSs and treated with IFN-αA/D (2000 U/ml) for 30 min at 48 h post-transfection. IFA was performed to detect NSs, the nuclei, and pSTAT1 or pSTAT2, shown in *red*, *blue*, and *green*, respectively. Each figure in the *red frame* and *blue frame* indicates the result of indirect IFA with anti-pSTAT1 and anti-pSTAT2, respectively. The a*rrow* and *arrowhead* indicate NSs-expressing and nonexpressing cells, respectively. Scale bar represents 50 μm. Representative results of IFA are shown. CRFK, Crandell–Rees feline kidney; HA, hemagglutinin; HEK293T, human embryonic kidney 293T cell line; IFA, immunofluorescence assay; IFN, interferon; NSs, nonstructural protein; STAT, signal transducer and activator of transcription.

We subsequently compared the effect of SFTSV NSs on the intracellular localization of phosphorylated STAT1 and STAT2 upon IFN-I stimulation in these cells using an IFA. As shown in Figure 6, SFTSV NSs formed characteristic cytoplasmic inclusion bodies regardless of the cell type. In the absence of NSs, both phosphorylated STAT1 and STAT2 were localized in the nuclei of all tested cells. We noted that there are three mechanisms by which phosphorylated STAT1 and STAT2 appear in NSs-expressing cells (Fig. 6) (1). Phosphorylated STAT1 appeared in the nucleus, whereas phosphorylated STAT2 did not (HEK293T [human] and Mpf [ferret] cells) (2). Both phosphorylated STAT1 and STAT2 were observed in the nucleus (NIH3T3 [mouse] and PK15 [pig] cells) (3). There was no nuclear staining for phosphorylated STAT1 and STAT2 (CRFK [cat] and A72 [dog] cells). These results indicate that NSs interferes with IFN-I-induced phosphorylation and nuclear translocation of human, canine, feline, and ferret STAT2 and canine and feline STAT1 but not murine and porcine STAT2 or human, murine, ferret, and porcine STAT1.

### Binding activity of NSs to each animal STAT1 and STAT2

SFTSV NSs suppress IFN-I signaling by interacting with STAT1 and STAT2 (21, 23). Therefore, we investigated the binding activity of NSs to STAT1 and STAT2.

**Figure 7:**
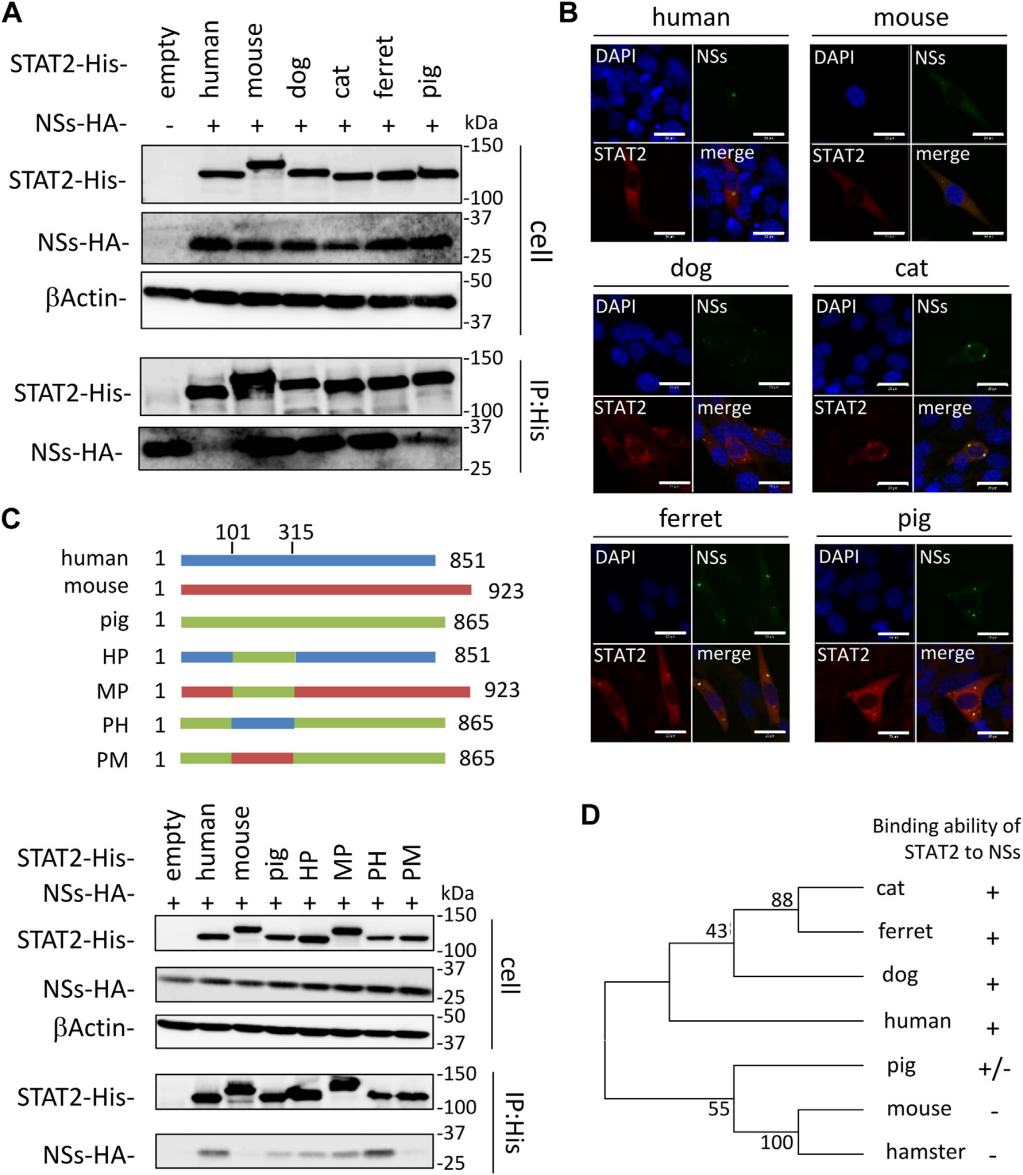
**Interaction of NSs with STAT2.***A*, NIH3T3 (mouse) cells were transfected with the expression plasmid for HA-tagged NSs and each of the His-tagged STAT2, and then co-IP assays were performed. *B*, colocalization of NSs with STAT2. NIH3T3 (mouse) cells were transfected with the expression plasmid for HA-tagged NSs and each of the His-tagged STAT2. IFA was also performed with NSs, STAT2, and the nuclei, shown in *green*, *red*, and *blue*, respectively. Scale bar represents 20 μm. *C*, identification of binding regions in porcine STAT2 to NSs. Schematic representation of the chimeric mutants of human, mouse, and pig STAT2 (*upper*). NIH3T3 (mouse) cells were transfected with the HA-tagged NSs and each of the His-tagged STAT2 chimeras, following which co-IP assays were performed (*lower*). *D*, the phylogenetic tree of the 101 to 315 region of each STAT2. This phylogenetic tree was constructed using the neighbor-joining method. The bootstrap values are indicated on each node. Representative results of Western blotting assays (*A* and *C*) and IFA (*B*) are shown. co-IP, coimmunoprecipitation; HA, hemagglutinin; IFA, immunofluorescence assay; NSs, nonstructural protein; STAT, signal transducer and activator of transcription.

We have previously shown that SFTSV NSs does not bind to murine STAT2 (24). Therefore, to examine the interaction of NSs with each animal STAT2, we performed co-IP assays using lysates from NIH3T3 (mouse) cells transfected with the expression plasmids for HA-tagged NSs and each animal STAT2. As shown in Figure 7*A*, NSs interacted with all animal STAT2, excluding mice. The interaction of NSs with STAT2 was also examined by subcellular colocalization of NSs and STAT2. The HA-tagged NSs expression plasmid was cotransfected with the expression plasmids for human, mouse, dog, cat, ferret, and pig STAT2 into NIH3T3 (mouse) cells, and subcellular localization of NSs and STAT2 was observed by IFA. Consistent with previous reports (21, 23), human STAT2 colocalized with NSs, whereas mouse STAT2 did not (Fig. 7*B*). NSs also colocalized with canine, feline, ferret, and porcine STAT2 (Fig. 7*B*).

**Figure 8:**
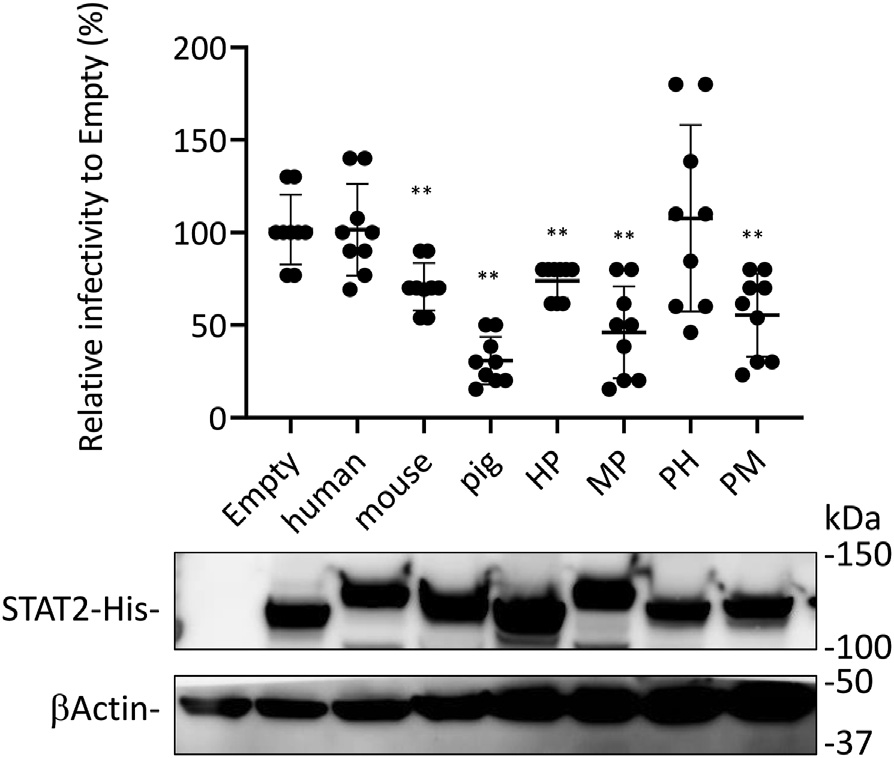
**Function of human, murine, porcine, and chimeric STAT2s in SFTSV infection.** SFTSV was inoculated into HEK293T (human) cells transfected with expression plasmid for each STAT2 at MOI 1. After 24 hpi, cells were treated with IFN-αA/D (500 U/ml) for 48 h and then collected culture supernatants. The SFTSV titer in culture supernatants was determined by focus-forming assay (*upper*). The protein expression was detected by Western blotting (*lower*). The assays were independently performed in triplicate. Values are the averages with SDs of data from nine results obtained from three experiments (n = 9). ∗∗*p* < 0.01, *versus* human STAT2. Each exact *p* value, average, and SD is shown in Table S4. HEK293T, human embryonic kidney 293T cell line; hpi, hours postinfection; IFN, interferon; MOI, multiplicity of infection; SFTSV, severe fever with thrombocytopenia syndrome virus; STAT, signal transducer and activator of transcription.

NSs levels in the IP eluents of porcine STAT2–expressing cells were lower than those in human, canine, feline, and ferret STAT2–expressing cells (Fig. 7*A*). Recently, we reported that the 101 to 315 region of human STAT2 is important for binding to NSs (24). Therefore, we hypothesized that the binding ability of porcine STAT2 to NSs is regulated by region 101 to 315 of porcine STAT2. To address this possibility, we prepared a chimeric STAT2 that replaced the 101 to 315 region of human STAT2 (HP-STAT2) or murine STAT2 (MP-STAT2) with that of porcine STAT2, which replaces the 101 to 315 region of porcine STAT2 with that of human STAT2 (PH-STAT2) or murine STAT2 (PM-STAT2). Interactions between NSs and chimeric STAT2 proteins were examined using a co-IP assay. The binding abilities of HP-STAT2 and MP-STAT2 to NSs were weaker than those of human STAT2 (Fig. 7*C*). In addition, the interaction of NSs with PM-STAT2 and murine STAT2 was not observed, whereas the binding ability of NSs to PH-STAT2 was comparable to that of NSs with human STAT2 (Fig. 7*C*). To further examine the relationship between the binding ability to NSs and the amino acid sequences in region 101 to 315, phylogenetic analysis was performed using the amino acid sequence of the 101 to 315 region in STAT2 of the animal species examined in this study and in hamster STAT2, which has been reported incapable of binding to NSs (24). There was a correlation between the amino acid sequences of the 101 to 315 region of STAT2 and the binding ability to NSs (Fig. 7*D*). To further examine the effect of the SFTSV replication by each chimeric STAT2 protein, SFTSV WT infected into HEK293T (human) cells transfected with expression plasmid for each STAT2 at MOI 1. At 24 hpi, cells were treated with IFN-αA/D (500 U/ml) for 48 h, and then the titer of SFTSV in culture supernatants was determined by focus-forming assay. The titer of SFTSV was significantly reduced by murine, porcine, HP, MP, PM STAT2, which poorly binds to NSs (Fig. 8). In contrast, human and PH STAT2, which bind to NSs, did not affect the SFTSV replication (Fig. 8). These results suggested that HP, MP, PM STAT2 as well as, murine and porcine STAT2, are not antagonized by SFTSV NSs. Our observations imply that NSs cannot interfere with IFN-I signaling in porcine cells and suppress the phosphorylation of porcine STAT2 because the binding activity of NSs to porcine STAT2 is weak.

**Figure 9:**
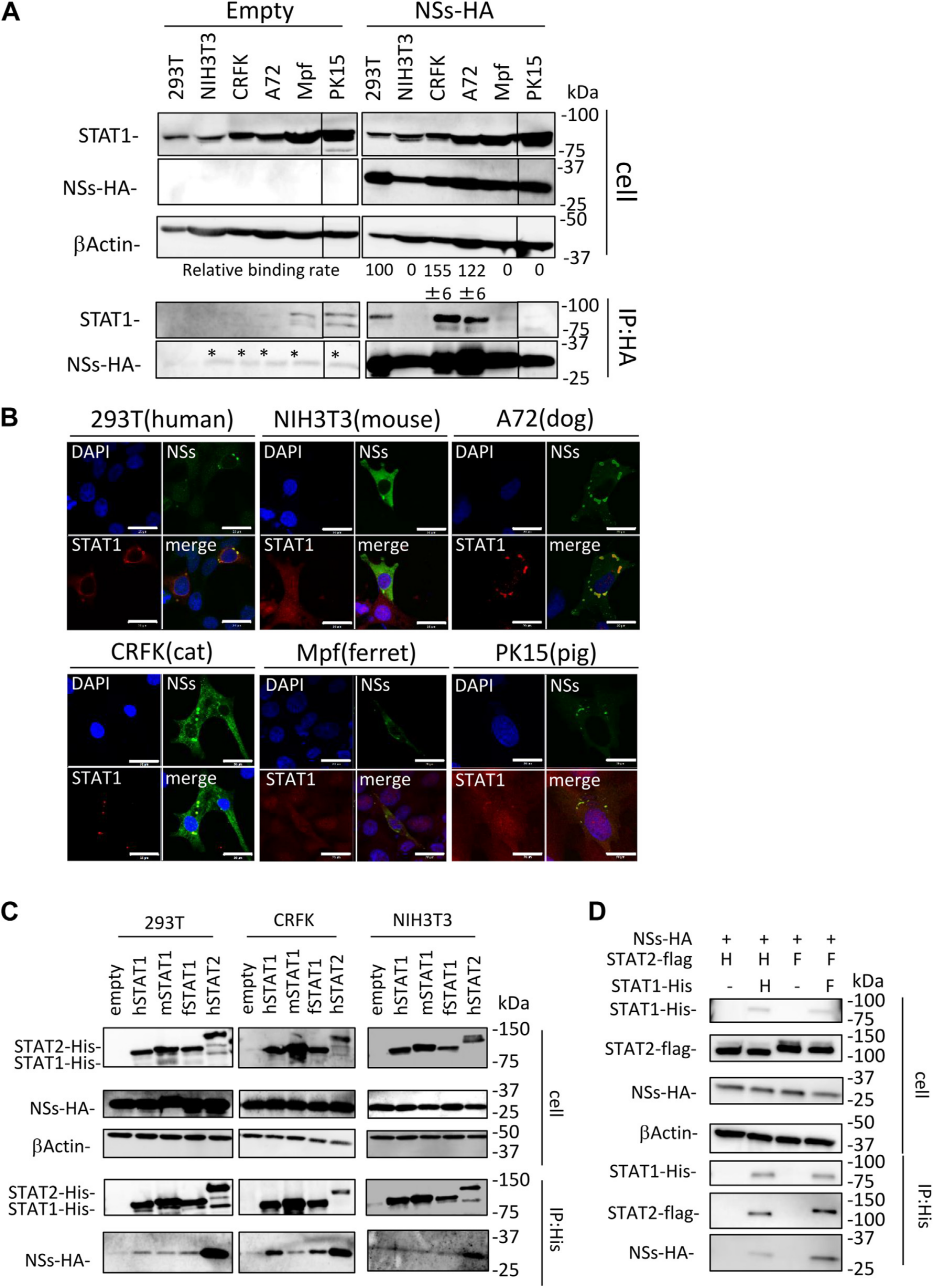
**Interaction of NSs with STAT1.***A*, HEK293T (human), NIH3T3 (mouse), CRFK (cat), A72 (dog), Mpf (ferret), and PK15 (pig) cells were transfected with the expression plasmid for HA-tagged NSs, and then co-IP assays were performed. The relative binding rates of NSs to human STAT1 are set at 100%. The assays were independently performed in triplicate. The data represent averages with SDs. The *asterisks* indicate the nonspecific bands. *B*, colocalization of NSs with STAT2. The expression plasmid for HA-tagged NSs was transfected to HEK293T (human), NIH3T3 (mouse), CRFK (cat), A72 (dog), Mpf (ferret), and PK15 (pig) cells. IFA was also performed with NSs, STAT1, and the nuclei, shown in *green*, *red*, and *blue*, respectively. Scale bar represents 20 μm. *C*, interaction of NSs with exogenous STAT1. HEK293T (human), NIH3T3 (mouse), and CRFK (cat) cells were transfected with the expression plasmid for HA-tagged NSs, His-tagged human STAT1 (hSTAT1), murine STAT1 (mSTAT1), feline STAT1 (fSTAT1), and human STAT2 (hSTAT2), and then co-IP assays were performed. *D*, Interaction activity of NSs to STAT1 and STAT2. The expression plasmid for HA-tagged NSs was transfected into NIH3T3 (mouse) cells with the expression plasmid for His-tagged human STAT1(H) or feline STAT1(F) and FLAG-tagged human STAT2 or feline STAT2, and then co-IP assays were performed. Representative results of Western blotting assays (*A*, *C*, and *D*) and IFA (*B*) are shown. CRFK, Crandell–Rees feline kidney; HA, hemagglutinin; HEK293T, human embryonic kidney 293T cell line; IFA, immunofluorescence assay; NSs, nonstructural protein; STAT, signal transducer and activator of transcription.

We further examined the interactions between NSs and STAT1 using a co-IP assay. The expression plasmid for HA-tagged NSs was transfected into HEK293T (human), NIH3T3 (mouse), CRFK (cat), A72 (dog), Mpf (ferret), and PK15 (pig) cells. As shown in Figure 9*A*, co-IP of STAT1 with NSs was observed in lysates of HEK293T (human), CRFK (cat), and A72 (dog) cells. We found that feline and canine STAT1 levels in IP eluents were 1.6 and 1.3 times higher than human STAT1 levels, respectively (Fig. 9*A*). In contrast, in NIH3T3 (mouse), Mpf (ferret), and PK15 (pig) cells, the interaction of STAT1 with NSs was not observed in the immunoprecipitation eluates. It is possible that the low efficiency of DNA transfection into NIH3T3 (mouse), Mpf (ferret), and PK15 (pig) cells prevented the detection of STAT1 in IP eluents. Therefore, we confirmed the interaction of STAT1 with NSs by subcellular colocalization of the proteins using indirect IFA (Fig. 9*B*). In HEK293T (human), CRFK (cat), and A72 (dog) cells, human, feline, and canine STAT1 colocalized with NSs, consistent with our co-IP assay (Fig. 9*B*). Colocalization of NSs with ferret and porcine STAT1 was also observed, although the interaction of NSs with ferret and porcine STAT1 was not observed in the co-IP assay (Fig. 9*B*). In contrast, NSs did not colocalize with murine STAT1 in NIH3T3 (mouse) cells (Fig. 9*B*). STAT1 was diffused throughout the cytoplasm in NSs-expressing HEK293T (human), Mpf (ferret), and PK15 (pig) cells, whereas it was completely localized to the cytoplasmic inclusion bodies in NSs-expressing CRFK (cat) and A72 (dog) cells. This difference may reflect the ability of NSs to bind to STAT1. These results suggest that NSs interacts with human, feline, canine, ferret, and porcine STAT1, and that the binding capacity of NSs to feline and canine STAT1 is stronger than that of NSs to human STAT1.

To understand the difference in the binding ability of NSs to STAT1 between humans and cats, we transfected the expression plasmid for HA-tagged NSs with the expression plasmid for human, murine, or feline STAT1 into HEK293T (human), CRFK (cat), and NIH3T3 (mouse) cells and then performed a co-IP assay. The expression plasmid for human STAT2 was used as a positive control. In HEK293T (human) and CRFK (cat) cells, NSs interacted with human STAT1 and feline STAT1 but not with murine STAT1 (Fig. 9*C*). However, NSs did not bind to STAT1 in NIH3T3 (mouse) cells. Unlike the experiment demonstrating the binding of NSs to endogenous STAT1 (Fig. 9*A*), the binding ability of NSs to exogenous human STAT1 was comparable to that of NSs to exogenous feline STAT1 (Fig. 9*C*). In addition, we confirmed that NSs interacted with human STAT2 in all the tested cells (Fig. 9*C*). These results suggest that the interaction between NSs and STAT1 is not direct. STAT1 forms a heterodimer with STAT2 (26). Therefore, we hypothesized that NSs interacts with STAT1 *via* STAT2 and that the binding abilities of feline STAT1 and STAT2 are stronger than those of human STAT1 and STAT2. To address this possibility, we performed co-IP assays using lysates from NIH3T3 (mouse) cells transfected with the expression plasmids for NSs, feline STAT1, human STAT1, feline STAT2, or human STAT2. As expected, the expression level of feline STAT2 in IP eluents was higher than that of human STAT2 (Fig. 9*D*). In addition, the co-IP of feline STAT1 with NSs was stronger than that of human STAT1 with NSs (Fig. 9*D*), suggesting that the interference of STAT1 phosphorylation by NSs depends on the binding strength of STAT2 and STAT1.

## Discussion

**Figure 10:**
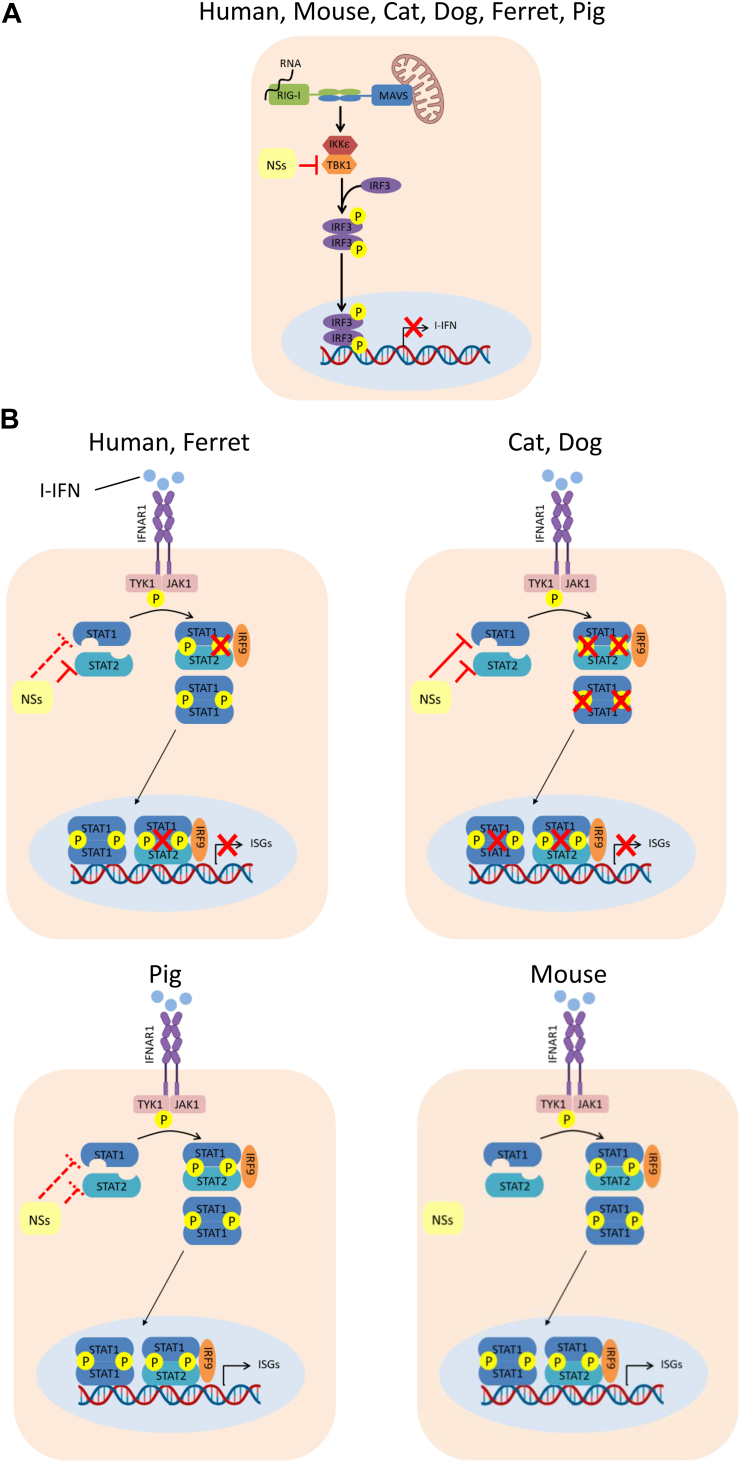
**Schematic diagram of NSs functionality in each animal.** Schematic summary of the differential mechanisms by which SFTSV NSs inhibit the (*A*) IFN-I induction and (*B*) signaling pathways among cells derived from various animal species. *Dashed red lines* indicate week interactions. *Solid red lines* indicate strong interactions. P indicates the phosphorylation. IFNAR, interferon-α/β receptor; IKKε, inhibitor of kappa B kinase-ε; JAK1, Janus kinase; MAVS, mitochondrial antiviral signaling protein; NSs, nonstructural protein; SFTSV, severe fever with thrombocytopenia syndrome virus.

In this study, we found that the interaction of NSs to TBK1 results in the inhibition of the IFN-I induction by viral infection, regardless of the cell type. In addition, we demonstrated that NSs suppressed the induction of ISG56 by IFN-I treatment in feline, canine, ferret, and human cell lines. In contrast, NSs cannot inhibit the IFN-I signaling pathway in porcine cells or murine cells. SFTSV is highly pathogenic in humans, cats, dogs, and aged ferrets but not in mice (1, 11, 14, 15, 24). In addition, it has been reported that anti-SFTSV antibodies have been detected in pigs, although no pigs have been diagnosed with SFTS (18). These findings suggest that the anti-IFN-I signaling activity of NSs, not the anti-IFN-I induction activity, determines the pathogenicity of SFTSV in each animal. In addition, we highlight the diversity in the dynamics of NSs to STAT1 and STAT2 in anti-INF-I activity of NSs among animals. These functions of SFTSV NSs in each animal are summarized in Figure 10. In this study, we indicated that the replication efficiency of SFTSV in NIH3T3 (mouse), MDTF (mouse), and PK15 (pig) cells was lower than that in HEK293T (human), CRFK (cat), FEA (cat), Cf2Th (dog), and A72 (dog) cells (Fig. 1). In addition, NSs inhibited IFN-I signaling in HEK293T (human), CRFK (cat), FEA (cat), Cf2Th (dog), and A72 (dog) cells but not in NIH3T3 (mouse), MDTF (mouse), and PK15 (pig) cells (Fig. 4). These results suggest that the differential suppression of IFN-I signaling by NSs in cells derived from different animal species correlates with the variable replication efficiency of SFTSV in these cells. Contrastingly, even though NSs inhibited IFN-I signaling in Mpf (ferret) cells, the growth efficiency of SFTSV in these cells was lower than that in HEK293T (human), CRFK (cat), FEA (cat), Cf2Th (dog), and A72 (dog) cells (Figs. 1 and 4*H*). The lower replication efficiency of SFTSV in Mpf (ferret) cells may be attributed to factors other than NSs, such as differences in the efficiency of host cellular proteins involved in viral replication. Moreover, SFTSV ΔNSs could not replicate in NIH3T3 (mouse), Mpf (ferret), and PK15 (pig) cells. The unknown restriction factors antagonized by NSs may be expressed in NIH3T3 (mouse), Mpf (ferret), and PK15 (pig) cells.

The induction level of ISG56 gene by IFN-I in Mpf (ferret) cells infected with SFTSVΔNSs was decreased from 44 hpi to 66 hpi (Fig. 4*H*). The details of its mechanism are unknown. However, the negative-feedback systems might be induced in Mpf (ferret) cells infected with SFTSVΔNSs at 66 hpi, since SFTSVΔNSs induced the IFN-I production at 18 hpi (Fig. 2*H*) (27).

In this study, the STAT2 phosphorylation was inhibited 48 h after SFTSV infection in Mpf (ferret) cells (Fig. 5*B*). In addition, the inhibition of the STAT2 phosphorylation was observed in NSs-transfected Mpf (ferret) cells (Fig. 5*A*). The results suggested that NSs suppresses the STAT2 phosphorylation in ferret cells if the expression of NSs is sufficient. Therefore, these results indicate that NSs inhibits the IFN-I signaling in ferret as well as human, cat, and dog.

We found that anti-STAT1 phosphorylation activity by NSs correlated with the binding strength of STAT2 and STAT1 (Figs. 5, 6, and 9*D*). However, it is unclear whether the binding strength between STAT2 and STAT1 directly contributes to the inhibition of STAT1 phosphorylation in NSs. Further analyses will be required to clarify this issue. In addition, we found that the binding activity of STAT1 to STAT2 differed between humans and cats (Fig. 9*D*). Although the STAT1 and STAT2 form the STAT1 homodimers, STAT2 homodimers, and STAT1–STAT2 heterodimers (26), the ratio of each dimer may differ among these species because of the different binding strength of STAT1 and STAT2. Previous study reported that heterodimers were more potent than homodimers in ISG induction by IFN-I (26). Therefore, the expression levels and kinds of ISGs induced by IFN-I possibly differ between humans and cats. In fact, the expression level of ISG56 induced by IFN-I was higher in CRFK (cat) and FEA (cat) cells than in HEK293T (human) cells (Fig. 4, *A*, *D*, and *E*).

As shown in Figure 8, the STAT2 of murine, porcine, HP, MP, and PM, which poorly binds to NSs, restricted the replication of SFTSV in HEK293T (human) cells. However, the restriction efficiencies of these STAT2 were not correlated with the binding ability of NSs to each STAT2. At present, we cannot explain this result, although this cause may be explained by the differences in the efficiency of ISGF3 formation with human IRF-9 among each STAT2.

The mortality rate associated with SFTS in humans varies with age. Mortality was lower in the young but higher in the elderly. In Japan, the reported median age of SFTS mortality is 81 years (https://www.niid.go.jp/niid/ja/sfts/3143-sfts.html). Similarly, aged ferrets (≥4 years) infected with SFTSV developed severe disease with high mortality, whereas young adult ferrets (≤2 years) did not demonstrate clinical symptoms or mortality (15). In contrast, cats infected with SFTSV showed severe thrombocytopenia with high mortality, regardless of age (28). However, the difference in susceptibility to SFTSV in young adults between humans, ferrets, and cats remains unknown. We previously reported that SFTSV causes severe thrombocytopenia in stat1^−/−^ mice (24). In this study, we found that the dynamics of STAT1 during SFTSV infection differ between humans and cats. These results suggest that STAT1 dynamics are correlated with disease pathogenicity in young adults during SFTSV infection. However, *in vitro* assay using cell lines alone are difficult to prove this relationship. Further analyses using *in vivo* experiments and/or the epidemiological studies will be required to clarify this issue.

SFTSV is transmitted to humans and animals through the bite of an SFTSV-infected tick (29). In addition, direct transmission from domestic cats to humans has been reported (11, 12, 13). In particular, veterinarians treating infected cats are at risk of SFTSV transmission (11, 13). Therefore, the prevention of SFTSV infection in companion animals, such as cats, may reduce the risk of transmission to humans. However, no effective vaccines are currently available to prevent SFTS. Recently, SFTSV ΔNSs was reported to be nonpathogenic to aged ferrets (30). In addition, aged ferrets inoculated with SFTSV ΔNSs prevented disease expression caused by SFTSV WT infection (30). In this study, we found that SFTSV ΔNSs did not inhibit IFN-I signaling in human, feline, canine, and ferret cells (Fig. 4). These results suggest that SFTSV ΔNSs can be used as live-attenuated vaccines in humans and many animals.

Taken together, our results serve to deepen our understanding of the importance as to anti-IFN-I activity of NSs in the pathogenicity of SFTSV to human and animals. In addition, these knowledges are helpful for vaccine development for human and animals.

## Experimental procedures

### Cell culture and virus

HEK293T (human) (CRL-11268; American Type Culture Collection [ATCC]), NIH3T3 (mouse) (CRL-1658; ATCC), MDTF (mouse), CRFK (cat) (CCL-94; ATCC), FEA (cat) (feline embryonic fibroblasts), Cf2Th (dog) (CRL-1430; ATCC), A72 (dog) (CRL-1542; ATCC), Mpf (ferret) (CRL-1656; ATCC), PK15 (pig) (CCL-33; ATCC), Vero 76 (CRL-1587; ATCC), and BHKT7/9 (a hamster kidney–derived BHK cell clone stably expressing T7 RNA polymerase) (31) cells were cultured in Dulbecco’s modified Eagle's medium (Sigma–Aldrich), supplemented with 10% heat-inactivated fetal calf serum and antibiotics (Thermo Fisher Scientific). The SFTSV YG1 strain, a field isolate from an SFTS patient in Japan, was kindly provided by Dr Ken Maeda, NIID. Virus stocks were prepared from the culture supernatants of Vero 76 cells.

### Focus-forming assay

Virus titration was performed by focus-forming assay using IFA with anti-SFTSV N antibodies, as previously described (32, 33). SFTSV titer was determined by counting the number of N-positive cells.

### Growth kinetics

Target cells were seeded in 12-well plates at 10^5^ cells per well. SFTSV or SFTSV ΔNSs (10^4^ FFU/ml) were inoculated into each well, and the plates were incubated for 1 h at 37 °C for viral adsorption. Culture supernatants were collected at 24, 48, and 72 h postinoculation. The titers of SFTSV or SFTSV ΔNSs were determined by focus-forming assay.

### Plasmid construction

HA-tagged SFTSV NSs, His-tagged human STAT2, and murine STAT2 expression plasmids were prepared as described previously (24). To prepare the expression plasmids for His-tagged canine, feline, ferret, and porcine STAT2, and FLAG-tagged human, murine, canine, feline, ferret, and porcine TBK1, the desired genes were amplified by RT–PCR using the primers listed in Table S5 from RNA extracted from HEK293T (human), NIH3T3 (mouse), Cf2Th (dog), CRFK (cat), Mpf (ferret), and PK15 (pig) cells, respectively. The expression plasmids for His-tagged human, feline, and murine STAT1, and FLAG-tagged human STAT2 and STAT2 chimeras (HP and MP) were constructed using an In-Fusion HD Cloning kit (Takara) with the primers listed in Table S5. Plasmids for the reverse genetics system of SFTSV were constructed as previously described (34, 35). Full-length L, M, and S complementary DNA (cDNA) segments were amplified from SFTSV (YG-1) viral RNA using RT–PCR. The cDNA segments were inserted into the T7 vector, between a T7 promoter and a hepatitis delta virus ribozyme sequence for the preparation of pT7-L, pT7-M, and pT7-S. The In-Fusion HD Cloning kit was used for cDNA insertion with the primers listed in Table S5. The open reading frame encoding RdRp and N was cloned into the pCAGGS vector using an In-Fusion HD Cloning kit with the primers listed in Table S5 for the preparation of pCAGGS-RdRp and pCAGGS-N. The NSs-deleted S segment (pT7-ΔNSs-S) was constructed using the KOD-mutagenesis Kit (Toyobo) with the primers listed in Table S5, using pT7-S as a template.

### Virus rescue

Recombinant SFTSV was generated as described previously (34, 35). BHK-T7/9 cells were cotransfected with pCAGGS-RdRp (0.1 μg), pCAGGS-N (0.5 μg), pT7-L (1 μg), pT7-M (1 μg), and pT7-S (1 μg) or pT7-ΔNSs-S (1 μg) using LT-1 (Mirus). Eight days after transfection, the virus-containing supernatant was transferred to Vero 76 cells and incubated at 37 °C for 4 days.

### Quantitative real-time RT–PCR

Quantitative real-time RT–PCR was performed as previously described using the primers listed in Table S5 (24). Relative mRNA levels were calculated using the 2^−ΔΔCT^ method with *GAPDH* mRNA as an internal control and are shown as relative fold changes normalized to untreated and uninfected control samples.

### Western blotting

Western blotting was performed as previously described using the following antibodies (24, 36): anti-HA (catalog no.: 18850; QED Biosciences, Inc), anti-His (catalog no.: 9F2; Wako), anti-FLAG (catalog no.: F7425; Sigma–Aldrich), anti-STAT2 (catalog no.: D9J7L; Cell Signaling Technology), anti-STAT1 (catalog no.: D19KY; Cell Signaling Technology), anti-STAT2 (phosphor Y690) (catalog no.: GTX50721; GeneTex), anti-STAT1 (phosphor Y701) (catalog no.: D4A7; Cell Signaling Technology), or anti-β-actin (catalog no.: AC-15; Sigma–Aldrich). The band intensities of pSTAT1, pSTAT2, STAT1, STAT2, and NSs-HA were quantified using ImageJ software (National Institutes of Health). The expression level of pSTAT1 or pSTAT2 was adjusted with the amounts of STAT1 or STAT2, respectively (Fig. 5, *A* and *B*). The STAT1 levels in IP eluents were adjusted with the amount of each NSs-HA (Fig. 9*A*).

### Transfection

All transfections were performed using LT-1 (Mirus) or Lipofectamine 3000 (Thermo Fisher Scientific), according to the manufacturer’s instructions. To investigate the localization and expression of pSTAT1 and pSTAT2, an expression plasmid for HA-tagged NSs was transfected into HEK293T, A72, CRFK, NIH3T3, Mpf, and PK15 cells. Forty-eight hours after transfection, the cells were treated with IFN-αA/D (2000 U/ml) (Sigma–Aldrich) for 30 min. To examine the binding or colocalization of TBK1 to NSs, the expression plasmid for HA-tagged NSs was cotransfected into HEK293T cells with the expression plasmid for FLAG-tagged human, murine, canine, ferine, ferret, or porcine TBK1. To examine the binding or colocalization of exogenous STAT2 to NSs, the expression plasmid for HA-tagged NSs was cotransfected into NIH3T3 cells with the expression plasmid for His-tagged human, murine, canine, ferine, ferret, or porcine STAT2. To examine the binding or colocalization of endogenous STAT1 with NSs, the expression plasmid for HA-tagged NSs was transfected into HEK293T, A72, CRFK, NIH3T3, Mpf, and PK15 cells. To verify the binding of exogenous STAT1 to NSs, the expression plasmid for HA-tagged NSs was cotransfected into HEK293T, CRFK, and NIH3T3 cells with the expression plasmid for His-tagged human, feline, or murine STAT1.

### Indirect IFA

All transfected cells were fixed using 4% paraformaldehyde–PBS (Wako), and the fixed cells were incubated in blocking buffer (5% goat serum and 0.3% Triton X-100 in PBS) for permeabilization and blocking. Cells were then treated with primary antibodies overnight at 4 °C and stained with secondary antibodies for 2 h at room temperature with 4′,6-diamidino-2-phenylindole (Roche) for visualization of nuclei. Images were acquired using an LSM780 microscope (Carl Zeiss).

### Co-IP assay

The co-IP assay was performed as previously described (24, 36). Cell lysates were mixed with magnetic beads conjugated to an anti-FLAG (FLA-1; MBL), anti-His (OGHis; MBL), or anti-HA monoclonal antibody (5D8; MBL) and incubated at 4 °C for 3 h or overnight. The magnetic beads were then washed with lysis buffer (25 mM Tris–HCl, 150 mM NaCl, 1 mM EDTA, and 1% Triton X-100) and wash buffer (50 mM Tris–HCl, 1% NP-40, 0.25% deoxycholic acid sodium salt, 150 mM NaCl, and 1 mM EDTA) and analyzed by Western blotting.

### Statistical analyses

The data are expressed as averages with SD, and statistically significant differences were determined using Student's *t* test. All statistical analyses were performed using Prism GraphPad 7 software (GraphPad Software, Inc). All exact *p* values, averages, and SD are shown in the Supporting information (Table S1–S4).

## Data availability

All data are included in the article.

## Supporting information

This article contains supporting information (21, 37, 38, 39, 40, 41).

## Conflict of interest

The authors declare that they have no conflicts of interest with the contents of this article.
